# PrecisionLymphoNet: Advancing Malignant Lymphoma Diagnosis via Ensemble Transfer Learning with CNNs

**DOI:** 10.3390/diagnostics14050469

**Published:** 2024-02-21

**Authors:** Sivashankari Rajadurai, Kumaresan Perumal, Muhammad Fazal Ijaz, Chiranji Lal Chowdhary

**Affiliations:** 1 School of Computer Science Engineering and Information Systems, Vellore Institute of Technology, Vellore 632014, India; sivashankari.r@vit.ac.in (S.R.); pkumaresan@vit.ac.in (K.P.); 2 School of IT and Engineering, Melbourne Institute of Technology, Melbourne, VIC 3000, Australia

**Keywords:** malignant lymphoma, chronic lymphocytic leukemia (CLL), follicular lymphoma (FL), mantle cell lymphoma (MCL), transfer learning, DenseNet201, Inceptionv3, Xception, ensemble technique

## Abstract

Malignant lymphoma, which impacts the lymphatic system, presents diverse challenges in accurate diagnosis due to its varied subtypes—chronic lymphocytic leukemia (CLL), follicular lymphoma (FL), and mantle cell lymphoma (MCL). Lymphoma is a form of cancer that begins in the lymphatic system, impacting lymphocytes, which are a specific type of white blood cell. This research addresses these challenges by proposing ensemble and non-ensemble transfer learning models employing pre-trained weights from VGG16, VGG19, DenseNet201, InceptionV3, and Xception. For the ensemble technique, this paper adopts a stack-based ensemble approach. It is a two-level classification approach and best suited for accuracy improvement. Testing on a multiclass dataset of CLL, FL, and MCL reveals exceptional diagnostic accuracy, with DenseNet201, InceptionV3, and Xception exceeding 90% accuracy. The proposed ensemble model, leveraging InceptionV3 and Xception, achieves an outstanding 99% accuracy over 300 epochs, surpassing previous prediction methods. This study demonstrates the feasibility and efficiency of the proposed approach, showcasing its potential in real-world medical applications for precise lymphoma diagnosis.

## 1. Introduction

Lymphoma, a form of hematological disorder, arises due to uncontrolled proliferation of lymphocytes, a subset of leukocytes. The lymphocytes, which are found in the blood and lymphatic tissues of the human body, have a crucial role in protecting the individual from various diseases. The lymphatic system comprises lymph nodes and lymphatic vessels responsible for draining fluid from bodily tissues and redirecting it to the circulatory system. Additionally, these structures aid in the removal of impaired, foreign, or aged cells. There are two types of lymphocytes, namely T and B. Both T and B lymphocytes reside in the lymph nodes. T cells have the ability to identify new antigens and transport them out of the body, while B lymphocytes produce antibodies. Lymphoma can be caused by any of these cells individually or in combination. As lymphocytes develop and form a mature lymph node, foreign lymphocytes will exceed the normal cells within the node. The symptoms and signs of the disease can vary depending on where the cancer originates, which body regions are affected, and the specific type of lymphoma [1].

**Figure 1:**
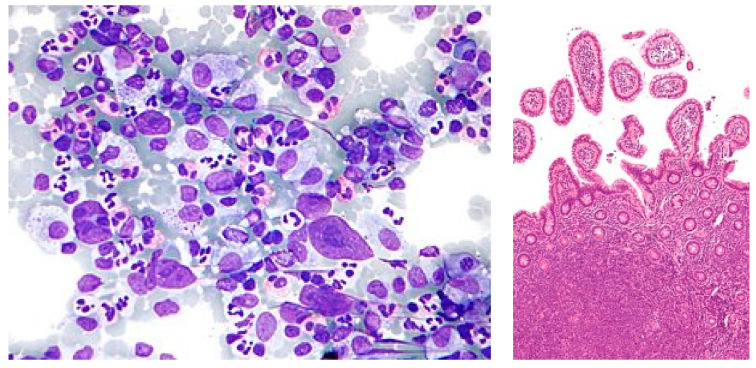
Hodgkin lymphoma and non-Hodgkin lymphoma (NHL).

Lymphoma typically presents itself in two distinct forms, namely Hodgkin lymphoma and non-Hodgkin lymphoma, as shown in Figure 1. The primary difference between these two variations of lymphatic cancer lies in the specific type of lymphocyte that is affected. While both Hodgkin lymphoma and non-Hodgkin lymphoma originate from B cells, only the latter is affected. Hodgkin lymphoma frequently originates in the upper regions of the body, such as the neck, chest, or armpits, whereas non-Hodgkin lymphoma can initiate in any lymph node throughout the body. Hence, there is a crucial need for diagnoses using automated techniques to overcome the existing challenges in malignant lymphoma [2].

Malignant lymphoma classification adopts several deep learning networks, which have played a significant role in recent years in analyzing Whole Side Images (WSIs) of pathological tissues of lymph nodes. This study focused on geometric, texture, and morphological analyses with feature-associated clinical and cytogenetic data [3].

The process of manually detecting malignant lymphoma is unfeasible. Potential solutions could be found using artificial intelligence models. Classification, segmentation, detection, and prediction are some of the applications of artificial intelligence models. Malignant lymphoma detection is recently popular and related works have been exhaustively analyzed and discussed [4]. 

This paper focuses on the objective of developing systems that are capable of predicting the specific type of malignant lymphoma based on histopathological samples that have been stained with Hematoxylin/Eosin (H + E). 

The primary contributions of this paper are as follows:

- Introduces a transfer learning CNN model comprising convolutional layers, pooling layers, and a fully connected layer tailored for multi-classification;
- A novel ensemble architecture, incorporating InceptionV3 and Xception, is proposed to enhance accuracy in lymphoma diagnosis, achieving an impressive 99% accuracy on multi-cancer datasets;
- Extensive testing is conducted on multi-class datasets from diverse sources, featuring chronic lymphocytic leukemia (CLL), follicular lymphoma (FL), and mantle cell lymphoma (MCL). This ensures the robustness and generalization of the proposed models;
- Features are extracted via convolutional layers, employing image scaling preprocessing, data augmentation, and spatial dimensionality reduction. The non-ensemble model, particularly compatible with Xception, outperforms other models with 97% accuracy and minimal validation loss on multiple cancer datasets.

The remaining sections of this work are as follows: Section 2 provides a summary of various relevant studies pertaining to the classification of malignant lymphoma. Section 3 describes the system’s architecture, specifically focusing on the pre-trained weights of VGG16, VGG19, DenseNet201, Inceptionv3, and Xception. Section 4 provides a malignant lymphoma image data description for the proposed architecture. Section 5 illustrates the proposed Non-Ensemble and Ensemble Transfer Learning architectures for malignant lymphoma classification. Section 6 discusses the performance evaluation metrics for training and testing the proposed system. Section 7 elaborates the experimental setup of the training and testing environment. Section 8 analyzes the results derived from the proposed system. Section 9 compares the performance of the proposed ensemble model with prior works. Lastly, Section 10 concludes the proposed system’s limitation and future work.

## 2. Related Works

This section provides an overview of several prior investigations that are pertinent to the identification of malignant lymphoma. All researchers intended to attain favorable outcomes via the implementation of distinct methodologies.

### 2.1. Machine Learning Methods for Malignant Lymphoma Classification

Capobianco et al. [1] proposed an ensemble model to find the Total Metabolic Tumor Volume (TMTV) calculated from F-labelled fluoro-2-deoxyglucose. The computed results showed that the model TMTV obtained 85% classification accuracy, 80% sensitivity, and 88% specificity in detecting lymphoma. Patil et al. [2] addressed the overlapping of blood cell image classification using Canonical Correlation Analysis (CCA). Several deep learning models were combined to perform the prediction of overlapping blood cell classification. A CNN was merged with many other deep learning models and computed the validation accuracy. The blood cells are mainly in two categories. Granular cell is the first category. The subcategories of granular cell are neutrophil, eosinophil, and basophil. Non-granular cell is the second category. Its subtypes are monocyte and lymphocyte. The combined deep learning models of CNN, VGG16, RNN, and LSTM obtained 89% accuracy. A CNN, InceptionV3, RNN, and LSTM combination achieved 91% accuracy. A CNN, ResNet50, RNN, and LSTM combination approach obtained 93% accuracy. A CNN, Xception, RNN, and LSTM combination obtained 95% accuracy. Tambe et al. [5] explored the automated morphometric analysis of cancer diagnosis using a deep learning technique. This method classified subtypes of lymphoma as chronic lymphocytic leukemia, follicular lymphoma, and mantle cell lymphoma and achieved an accuracy of 97.33%. Steinbuss, G et al. [6] demonstrated that an EfficientNetB3 deep learning model is suitable for the classification of tumor-free lymph nodes and tumor lymph cells of CLL and DLBCL. The EfficientNetB3 secured 95.56% accuracy on classification above lymph categories. El Achi et al. [7] proposed the prediction and diagnosis of lymphoma using CNN modeling. The CNN modeling is used to build diagnostic models into four types, namely benign lymph nodes, diffuse large B-cell lymphoma, Burkitt lymphoma, and lymphoma small cells. The obtained validation accuracy of the CNN modeling on diagnosis of the above five categories was 95%. Shrot et al. [8] distinguished different types of brain tumors via an experimental study of 141 patients (41 glioblastomas, 38 metastatic tumors, 50 meningioma, and 12 primary CNS lymphomas) using basic and advanced MRI sequencing and obtained better accuracy than other existing methods. Miyoshi et al. [9] determined malignant lymphoma from histopathological images by using an ensemble approach. The ensemble model combines prediction results of each three deep learning models and an averaging approach is used to predict the final malignant lymphoma. Sibille et al. [10] evaluated lung cancer and lymphoma using a deep convolutional neural network (CNN) which classified the 18F-FDG PET/CT images into cancer patients or not.

Gaidano et al. [11] developed an immune phenotypic prediction model which consisted of multiple decision tree approaches for detecting B-cell non-Hodgkin lymphoma in blood cell images. Three different decision trees are built from the features present in the dataset. Since the selected decisions are very suitable for lymphoma detection for model 2, model 2 obtained 92% accuracy, model 1 obtained 87%, model 3 obtained 89%, and model 4 obtained 87%. The author demonstrates the strong discriminating power of MIB1 and Bcl2, whose integration in the predictive model significantly increases the performance of the algorithm. The method measured the potential utility of some nonconforming markers in the B-NHL classification. The FC markers do not qualify as positive or negative under fixed thresholds, but rather they are correlated with different B-NHLs depending on their expression level.

Ijaz et al. [12] presented a cervical cancer prediction model for the early detection of cervical cancer using Random Forest classifier models such as iForest-SMOTETomek and iForest-SMOTE approaches. This model outperformed when compared to other existing approaches. 

### 2.2. Deep Learning Methods for Malignant Lymphoma Classification

Zhao et al. [13] distinguished diseased samples from healthy samples using a CNN model. They classified seven subtypes of adult B-cell tumors such as chronic lymphocytic leukemia, marginal zone lymphoma, mantle cell lymphoma, prolymphocytic leukemia, follicular lymphoma, hairy cell leukemia, and lymphoma. The reliability of the classification was 70% of appeals with 95% confidence. Sheng, B., Zhou et al. [14] used a large number of blood cell datasets, which contained lymphoma cells, lymphocytes, blast cells, and an annotation file of each image file. The authors determined the final model by testing the performance of a combination of different training methods and networks on this dataset and testing its performance against a brand-new dataset. The final results of the test found that the lymphoma detection rate was greater than 95%. Lippi et al. [15] developed a multi-version learning model using support vector machine with texture features. The result showed the detection of Hodgkin lymphoma was more than 90% accurate. Zhang et al. [16] presented the classification of NHL subtypes based on the fusion of transfer learning (TL) to detect non-Hodgkin lymphoma and its subtypes from digital pathological images. For feature extraction, a Principal Component Analysis (PCA) approach was used. 

Rajpurohit et al. [17] diagnosed acute lymphoblastic leukemia blood cancer. The detection of this type of cancer is performed manually by looking at a patient’s blood sample under a microscope and performing a variety of tests. The authors used blood images and applied various classifiers such as CNN, FNN, SVM, and KNN to automate the above-mentioned manual work. Brancati et al. [18] presented a deep learning approach with specific parameters for cancer detection and classification. They used fusion Net encoder for image segmentation and the reconstruction approach was adapted for cancer detection and histological image classification. They conducted a comparison with conventional approaches. Biccler et al. [19] presented the predictive performance of prognostic scores in various types of malignant lymphoma and plotted the obtained score results.

Srinivasu et al. [20] proposed a deep-learning-based MobileNet V2 and Long Short Term Memory (LSTM) model for detecting skin disease from an image of the region of interest at an early stage, assisting physicians in predicting skin conditions efficiently and minimizing further complications.

### 2.3. Transfer Learning Methods for Malignant Lymphoma Classification

We analyzed a pre-trained weight transfer learning model for malignant lymphoma classification. The findings achieved in this study are quite promising and the performance values are represented in Table 1.

## 3. Materials and Methods

Lymphomas can be categorized into two primary groups. These groups consist of non-Hodgkin lymphoma and Hodgkin lymphoma. The non-Hodgkin type encompasses three key subgroups and is more malignant compared to the Hodgkin type. These subgroups include chronic lymphocytic leukemia (CLL), mantle cell lymphoma (MCL), and follicular lymphoma (FL). The most dangerous and persistent form of leukemia within this group is chronic lymphocytic leukemia (CLL). The proposed system utilized Convolution Neural Network (CNN) neural network algorithms to train the models, make predictions, compare the results, and determine the most accurate outcome.

### 3.1. Visual Geometry Group16 (VGG16)

The Visual Geometry Group VGG-16 is comprised of a total of 16 layers. Among these layers, there are 3 fully linked layers and 13 convolutional layers. The max-pooling layers within the network possess a filter size of 2 × 2, with a stride of 2 pixels. On the other hand, each individual convolutional layer has a filter size of 3 × 3, with a stride of 1 pixel. This network is designed to receive an RGB image with an input size of 224 × 224 pixels. The output layer of the network consists of 1000 units, each corresponding to one of the 1000 Image Net classes. The VGG-16 architecture effectively utilizes the combination of depth and tiny filters (3 × 3) in its convolutional layers, enabling it to capture complex features within images [29].

### 3.2. Visual Geometry Group19 (VGG19)

The Visual Geometry Group VGG-19 consists of a total of 19 layers, comprising 16 convolutional layers and 3 fully linked layers. The additional convolutional layers in VGG-19 are specifically designed to capture more complex features present in the input images, thereby improving its accuracy for image recognition. Similar to the VGG-16, the VGG-19 consists of 2 × 2 max-pooling layers. These layers have a stride of 2 pixels and small 3 × 3 filters in all convolutional layers. The stride of these filters is set to 1 pixel. The final layer of the network consists of 1000 units, aligning with the 1000 ImageNet classes and the input to the network is RGB image with a size of 224 × 224 pixels [30]. 

### 3.3. DenseNet201

DenseNet-201 primarily addresses disappearing gradients in deep neural networks via the implementation of feed-forward networks linking each layer to all other layers. This dense connectivity approach decreases the required parameters and enables the reuse of features across layers, thus improving the performance of the model. DenseNet-201 comprises 201 levels, which consist of multiple dense blocks that are connected by transition layers. Each dense block consists of a series of convolutional layers with a predetermined number of filters, followed by a bottleneck layer that reduces the number of channels. The input for each layer in a dense block is the concatenated feature maps from all previous layers. This transition layer includes a batch normalization layer, a 1 × 1 convolution layer for dimensionality reduction, and a max-pooling layer [31]. The input for DenseNet-201 consists of an RGB image which has a dimension of 224 × 224 pixels. The output layer of DenseNet-201 is composed of 1000 units that correspond to the 1000 classes in the ImageNet dataset. 

### 3.4. Inception v3

The primary objective of Inception v3 is to augment the precision and effectiveness of the Inception architecture via the implementation of significant modifications. Notably, one of the most prominent modifications is the integration of batch normalization, which decreases the internal covariate shift and expedites the process of training. Furthermore, Inception v3 adopts factorized 7 × 7 convolutions rather than 7 × 7 convolutions to minimize the quantity of parameters within the network. Inception v3 comprises a pooling layer and multiple parallel convolutional layers having diverse filter sizes of 1 × 1, 3 × 3, and 5 × 5. The outputs of these parallel layers are merged and provided to the subsequent layer. To enhance the network’s ability to learn more distinctive features, auxiliary classifiers are integrated into the model and placed into the middle of the network. The input to the Inception v3 network is an RGB image with a size of 299 × 299 pixels, which is significantly larger than the original Inception design’s input size. The output layer of the network consists of 1000 units, corresponding to the 1000 classes in the ImageNet dataset [32].

### 3.5. Xception

Xception uses depth-wise separable convolutions as a standard alternative for the basic convolutional layers found in the Inception architecture. A depth-wise separable convolution is a two-step convolution process that first implements a spatial convolution on each input channel separately, and subsequently performs a point-wise convolution to combine the outputs of the spatial convolutions. This approach increases the power of the convolutional layers with a smaller number of parameters and computations. To optimize the effectiveness of the model, Xception combines both skipping connections and residual connections in addition to multiple depth-wise separable convolutional layers. The input to the Xception network is an RGB image with dimensions of 299 × 299 pixels, while the output layer comprises 1000 units that map to the 1000 classes in the ImageNet dataset [33].

## 4. Dataset Description

Lymphoma has become the seventh most common cancer expected to occur and the ninth most common cause of cancer death in both males and females. However, pathological diagnosis as the main diagnostic method is time-consuming, expensive, and error-prone. Most of the researchers use the lymphopath database of the cancer research institute. The analysis is performed with multiple datasets such as ImageNet, PASCAL VOC, and MS COCO Dataset kaggle multicancer WSI images. The researchers use microscopic blood image datasets with samples of leukocytes and modified by their type’s lymphoma. Lymphoma has three categories such as CLL (chronic lymphocytic leukemia), FL (follicular lymphoma), and MCL (mantle cell lymphoma). CLL is one of the bone marrow and blood cancer types. The bone marrow contains a soft tissue from which blood cells are generated. FL is the second type of cancer in the lymph nodes, bone marrow, and organs. FL is naturally indolent and its cancer cell development is slow in this category. The MCL cancer type starts with white blood cells in lymph nodes.

**Figure 2:**
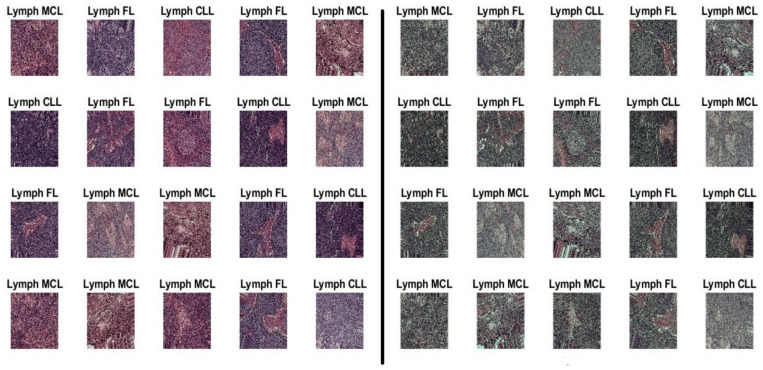
The malignant lymphoma image samples of CLL, FL, and MCL.

This system evaluated histopathological images of the multiple datasets of malignant lymphoma. The non-ensemble model of pre-trained network will be fine-tuned using benchmarked datasets. There are two different datasets used in this paper. Both the datasets are downloaded from the kaggle repository. The first dataset is downloaded from the URL https://www.kaggle.com/datasets/andrewmvd/malignant-lymphoma-classification (accessed on 12 December 2023) and the dataset size is 374. In this work, a total 334 TIF-formatted samples are used for training and 40 samples are used for testing the framework. Within the 334-training dataset are 109 samples of CLL, 124 samples of FL, and 109 samples of Myelofibrosis of MCL [34]. The test data are split into 12 samples of CLL, 15 samples of FL, and 13 samples of MCL. The convolutional neural network is initialized with RGB images of size 240 × 240 pixels. Finally, the most effectively trained models, the InceptionV3 and Xception models, are combined as an ensemble architecture for diagnosing lymphoma. The second dataset used in this paper consists of 15,000 images and the URL is https://www.kaggle.com/datasets/obulisainaren/multi-cancer (accessed on 12 December 2023). The dataset contains three equal parts of 5000 for CLL, 5000 for FL, and 5000 for MCL. The test data are split into 1029 samples of CLL, 962 samples of FL, and 1009 samples of MCL. All dataset images were in the JPEG file format with a size of 512 × 512 pixels. The dataset employed in this investigation is shown in Figure 2.

## 5. Proposed Non-Ensemble and Stacked Ensemble Transfer Learning Architecture for Malignant Lymphoma Classification

**Figure 3:**
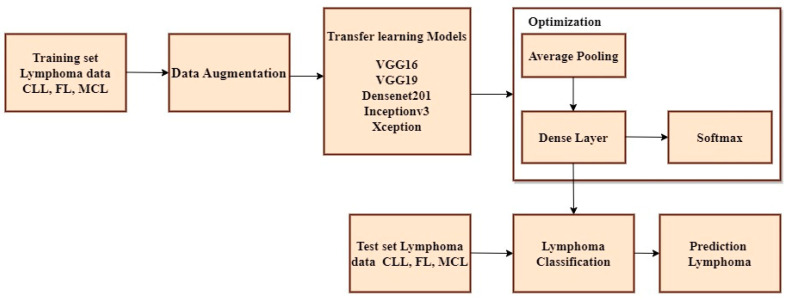
Non-ensemble transfer learning architecture.

The non-ensemble model is trained using VGG16, VGG19, DenseNet201, Inceptionv3, and Xception as shown in Figure 3. 

A Convolutional Neural Network (CNN) is a type of neural network architecture used for tasks such as image classification, object detection, and other computer vision applications. CNNs are specifically designed to identify patterns of input images via the utilization of trained convolutional layers. The CLL, FL, and MCL image datasets are imported from Kaggle and multiple platforms and fed as an input block. Several pre-processing methodologies such as data augmentation, scaling, image formatting, and RGB conversion are used to create and train the model. Initially, color inversion is performed on the picture dataset using RGB conversion. Subsequently, image formatting was undertaken, where the file extension of the image dataset was modified from JPEG to TIFF. Furthermore, image scaling is done to the size of 224 × 224 pixels. Finally, data augmentation is performed using flipping, rotating, cropping, and padding of the image [35]. The Convolutional Neural Network (CNN) comprises several layers, such as convolutional layers, pooling layers, and fully connected layers. In a typical CNN, the first few layers are convolutional layers that extract features from the input images. These layers employ multiple filters to the input data, and the output of each filter is a feature map that represents the presence of a specific element in the data. Pooling layers are often utilized after convolutional layers to simplify the spatial dimensionality of feature maps. The most widely used type of pooling is max pooling, which reduces the size of the feature map by selecting the highest value within each spatial region. The resulting data are transformed into a compressed form and transmitted to one or more completely interconnected layers, which execute the categorization, following a series of repeated convolutional and pooling stages. The Softmax activation function is applied to the outcome of the final fully interconnected layer to obtain the probability distribution across all potential classes. The initial input to the Convolutional Neural Network (CNN) consists of an image represented as a matrix of pixel values. Subsequently, multiple convolutional layers are applied to this image. Each convolutional layer uses a set of trainable filters on the input image, resulting in a set of feature maps. These feature maps represent the activation of the filters at various spots across the input image. To add non-linearity and to increase the efficacy of the model, each feature map undergoes a non-linear activation function, such as Rectified Linear Unit (ReLU). Following this, the feature maps are subjected to pooling layers that down-sample them by selecting the highest or average value within each spot of the map. This process reduces the spatial dimensionality of the feature maps and improves the model’s robustness against minor input variations. 

**Figure 4:**
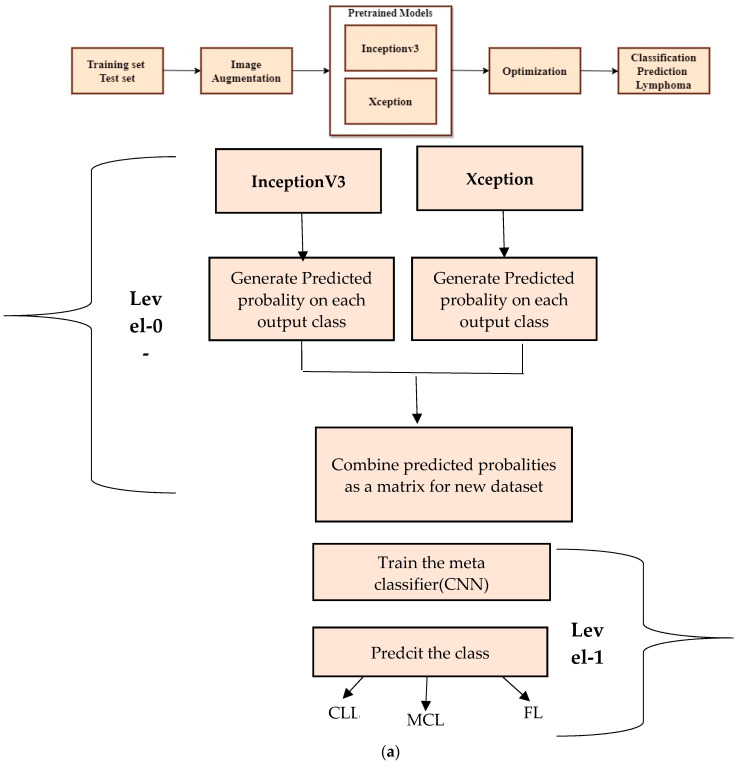
Ensemble transfer learning architecture. (**a**) Proposed method of Stacked Ensemble Technique steps.

The output of the final pooling layer is then flattened and applied through one or more fully connected layers for classification. Typically, a SoftMax layer is employed as the top layer, generating a probability distribution for all possible classes. The class with the highest probability is chosen as the prediction. To determine the optimal values for the filter weights and biases, the model is trained using a dataset of labelled images and a stochastic gradient descent optimization algorithm. For testing the non-ensemble model, 342 TIF-formatted samples are used for training and 40 samples are used for testing the framework. The test data are split into 12 samples of CLL, 15 samples of FL, and 13 samples of MCL. Inceptionv3 and Xception attain maximum efficiency when compared to other pre-trained models. To improve the accuracy more than one algorithm is essential. Thus the proposed approach has used Inceptionv3 and Xception advanced deep neural network models to enhance the prediction accuracy. Figure 4a shows a step-by-step approach for stacking the ensemble approach to classify lymphoma cells. 

The stacked ensemble model is trained and tested using a second dataset. The multi-class images are divided into three equal parts of 5000 for CLL, 5000 for FL, and 5000 for MCL. The 15,000 samples are used for training and 3000 samples are used for testing the model. The test data are split into 1029 samples of CLL, 962 samples of FL, and 1009 samples of MCL. All dataset images were in the JPEG file format with a size of 512 × 512 pixels. The advanced deep learning models such as Xception and InceptionV3 are the best suited models for image classification. Thus, the proposed method deployed these two models as the level-0 classifier models. These two advanced neural network architectures are trained using training samples and generate a new dataset for the second-level classification. 

## 6. Evaluation Metrics for Proposed Models

The Mean Absolute Error (**MAE**) shown in Equation (1) is a widely used metric for assessing the efficacy of a predictive model. It quantifies the average absolute difference between the predicted values and the actual values of the target variable.

(1) $$MAE=\frac{1}{n}\sum_{1}^{n}\left(ytrue-ypredicted\right)$$

The Mean Squared Error (**MSE**), as depicted in Equation (2), quantifies the average of the squared difference between the predicted values and the original values of the target variable.

(2) $$MSE=\frac{1}{n}\sum_{1}^{n}{\left(ytrue-ypredicted\right)}^{2}$$

The Mean Absolute Percentage Error (**MAPE**), as shown in Equation (3), is used as a quantitative measure for evaluating the accuracy of a forecasting model. This measure is derived from computing the mean percentage difference between the actual values and the predicted values.

(3) $$MAPE=\frac{1}{n}\sum_{1}^{n}\left(\frac{ytrue-ypred}{ytrue}\right)\;*\;100$$

Accuracy, precision, recall, and F1 score are performance metrics commonly used in classification problems to evaluate the performance of a model. The measure of accuracy is derived from the ratio of correctly classified points to the total number of points, as expressed in Equation (4).

(4) $$Accuracy=\frac{TP+TN}{TP+FP+TN+FN}$$

where **TP** is True Positive, **TN** is True Negative, **FP** is False Positive, and **FN** is False Negative.

Precision refers to the fraction of correctly categorized instances out of the overall classified instances, as shown in Equation (5).

(5) $$Precision=\frac{TP}{TP+FP}$$

The recall or sensitivity can be defined as the ratio of correctly classified instances to the total number of instances classified, as shown in Equation (6).

(6) $$Sensitivity\;or\;Recall=\frac{TP}{TP+FN}$$

The F1 score can be defined as the harmonic mean of precision and recall, as expressed in Equation (7).

(7) $$F1\;Score=\frac{2\;*\;Precision\;*\;Recall}{Precision+Recall}$$

Specificity measures the number of instances of true negatives that are correctly identified by the model, as shown in Equation (8).

(8) $$Specificity=\frac{TN}{TN+FP}$$

In the context of classification models, True Positives (**TPs**) refer to the instances that are truly positive and have been accurately classified as positive by the model. False Positives (**FPs**), on the other hand, denote the instances that are actually negative but have been erroneously classified as positive by the model. Similarly, True Negatives (**TNs**) represent the instances that are genuinely negative and have been correctly classified as negative by the model. Lastly, False Negatives (**FNs**) pertain to the instances that are truly positive but have been wrongly classified as negative by the model [36].

## 7. Experimental Setup

The Kaggle Framework was used to train the experiment using an Intel i9-12900 2.4 GZ 30 MB 16 Cores 64 W CPU (Intel, Santa Clara, CA, USA) and NVIDA RTX A2000 Graphics system (NVIDA, Santa Clara, CA, USA). The proposed stacked ensemble method is implemented in the Kaggle notebook.

## 8. Performance Analysis and Discussion

In this particular section, our primary focus is on the multiple source datasets employed throughout the training and testing phases of five different CNN models such as VGG16, VGG19, DenseNet201, Inceptionv3, and Xception. An ensemble architecture is proposed to increase accuracy using InceptionV3 and Xception. Training and testing are performed for the ensemble architecture using a multi-cancer lymphoma Kaggle dataset. Subsequently, we discuss the outcomes of the proposed ensemble learning model on the mentioned CLL, FL, and MCL datasets. The pre-trained models are trained and tested at a learning rate of 0.001. The proposed model has used the kaggle notebook for the implementation. The CLL, FL, and MCL datasets are available in individual directories. The directories are loaded into the kaggle user environment [37]. To assign target values for each image to train the deep learning models, python library label.index(foldername) and other predefined python library functions are used.

### 8.1. Performance Evaluation of VGG16 Model

**Figure 5:**
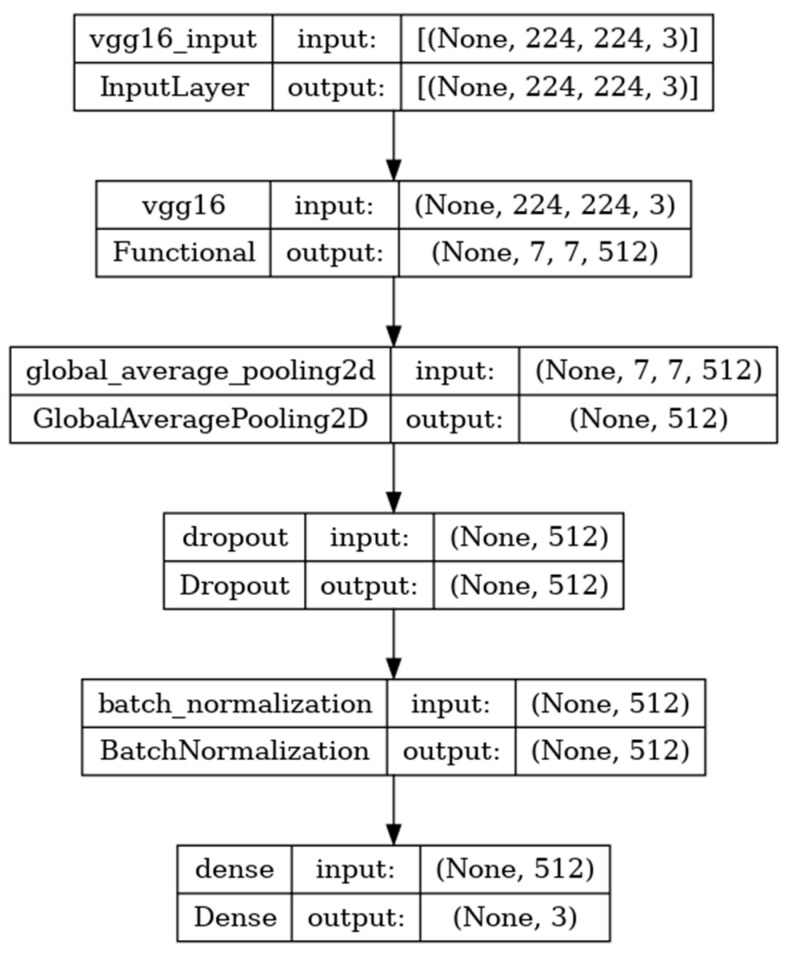
VGG16 architecture summary.

The VGG16 model is sequentially composed of 16 deep convolutional layers. The model shown in Figure 5 is developed with the input, functional, and output layers. The pooling layer is a fixed operation with no weighting factor [38].

**Figure 6:**
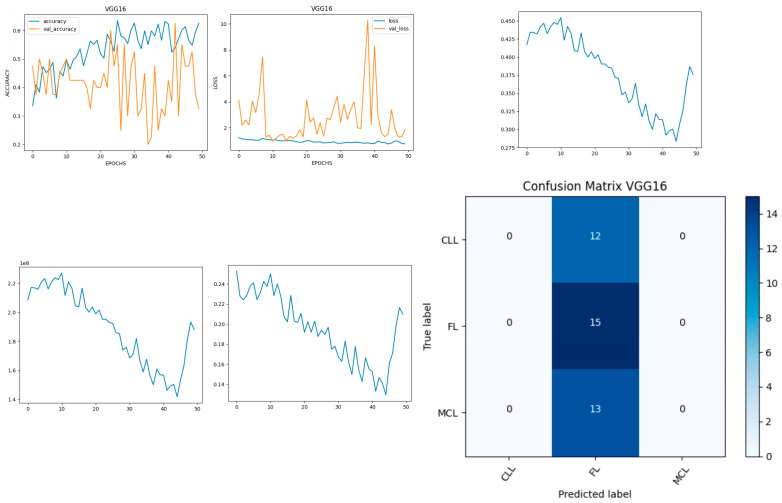
Accuracy, loss, **MAE**, MSE, MAPE, and confusion matrix of VGG16.

Figure 6 shows the experimental results obtained using the VGG16 model. The number of epochs is set to 50. In VGG16, the training accuracy is gradually increased from 35% to 60%. The validation accuracy increased, decreased, and then attained a maximum 60% at the 45th epoch. The validation loss is around 4 in the 1st epoch and increases until the 8th epoch to 7.8, but again decreases and attains a minimum at the 10th epoch to 1. The validation loss is greater than the training loss. Thus, the model is over fitted. The capability of the VGG16 **MAE** value is 0.0281, the MSE value is 0.0045, and the MAPE value is 0.3310 for the test data [39].

The confusion matrix shows that 15 samples are correctly predicted out of the total 40. Thus, overall accuracy is 38%. The macro average precision is the simple arithmetic average of the precision of all the class and the value obtained is 0.12. The weighted average precision obtained is 0.14. The higher F1 score suggests better model performance and the FL class has a maximum of 0.55. The F1 scores of all classes are balanced between precision and recall as shown in Table 2.

### 8.2. Performance Evaluation of VGG19 Model

The VGG-19 model has a convolutional neural network of 19 deep layers.

**Figure 7:**
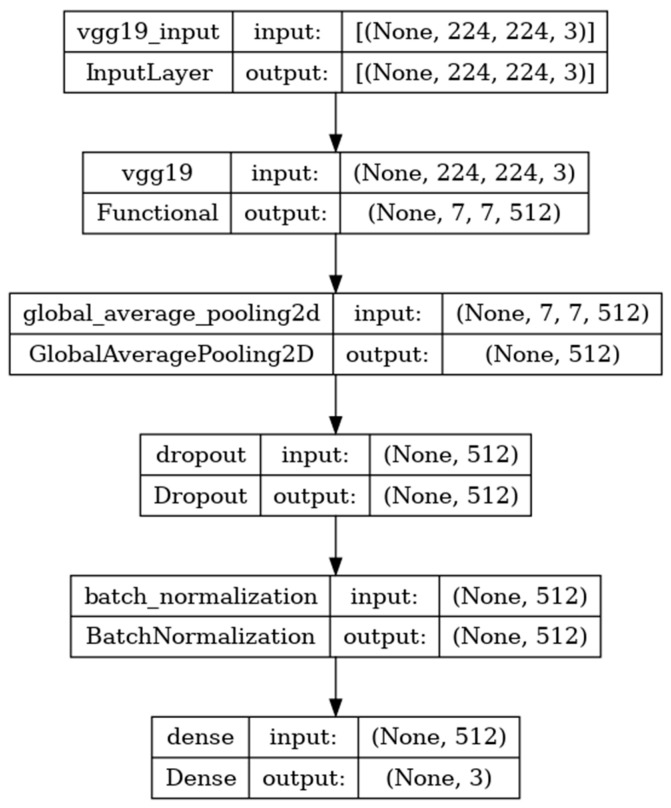
VGG19 architecture summary.

**Figure 8:**
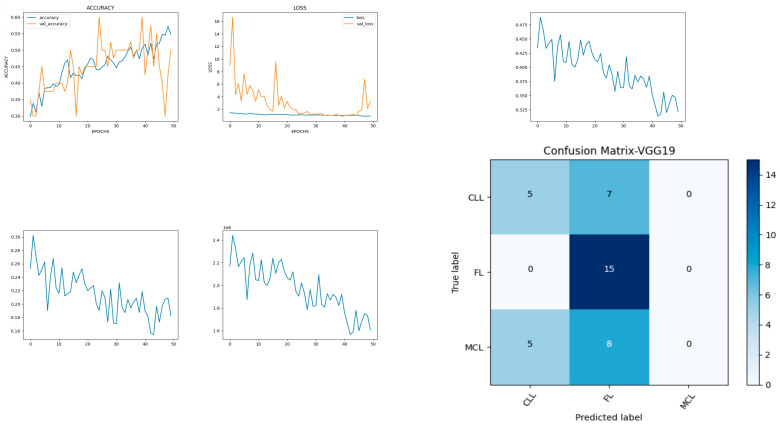
Accuracy, loss, **MAE**, MSE, MAPE, and confusion matrix of VGG19.

The model developed is shown in Figure 7 and includes the input, functional, and output layers. The pooling layer is a fixed operation with no weighting factor [40]. Figure 8 shows the experimental results obtained using the VGG19 model. The number of epochs is set to 50. In VGG19, the training accuracy is gradually increased from 30% to 55%. The increasing and decreasing validation accuracy attains a maximum of 55% on the 48th epoch. The validation loss is around 9 in the 1st epoch and instantly increases to 16. The increasing and decreasing validation loss attains a minimum value at epoch 45. The validation loss is greater than the training loss. Thus, the model is over fitted. The capability of the VGG19 **MAE** value is 0.30, the MSE value is 0.15, and the MAPE value is 1.2 for the test data [41]. The confusion matrix shows that 20 samples are correctly predicted out of a total 40. Thus, overall accuracy is 50%. The macro average precision of all the multiple classes is 0.33. The weighted average precision obtained is 0.34. The higher F1 scores among the multiple classes improve the model performance and the FL class has a maximum of 0.67. The F1 scores of all classes are balanced between precision and recall as shown in Table 3.

### 8.3. Performance Evaluation of DenseNet201 Model

**Figure 9:**
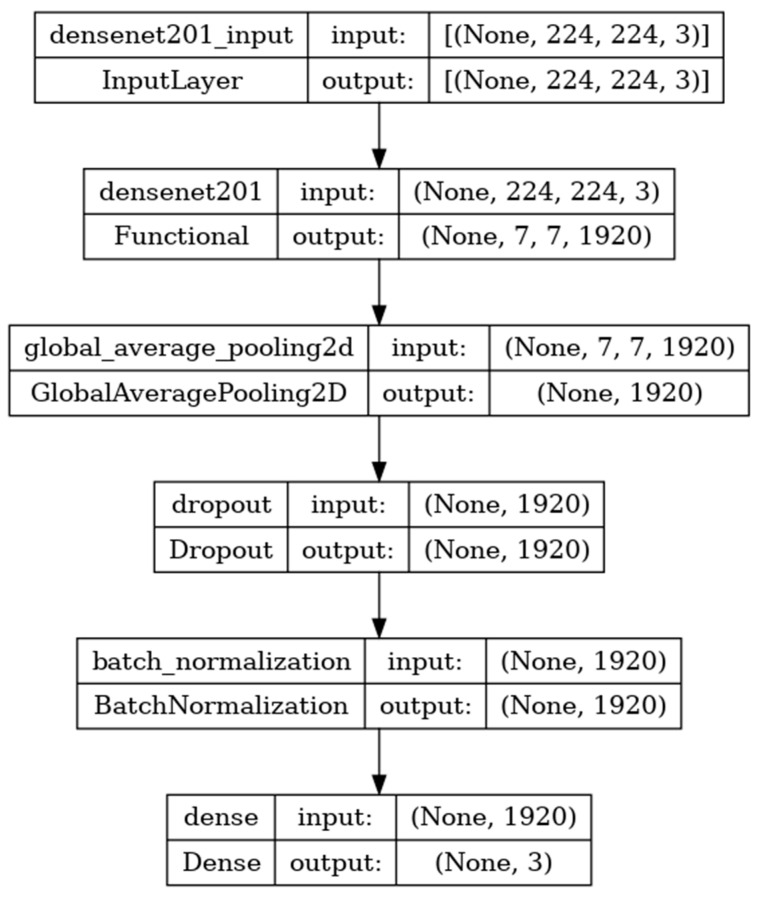
DenseNet201 architecture summary.

**Figure 10:**
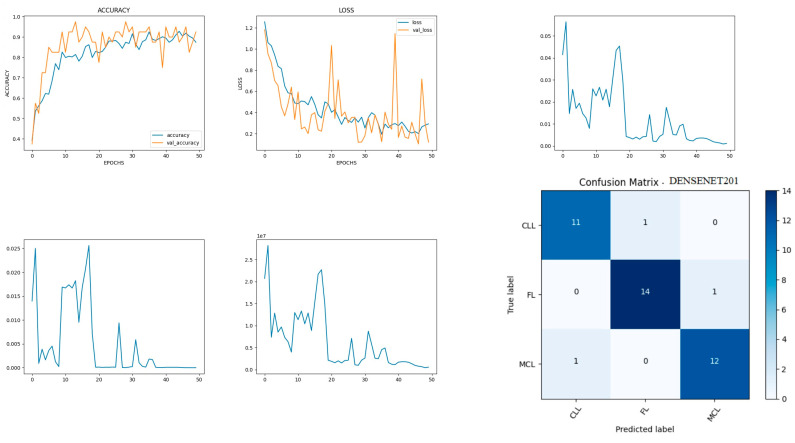
Accuracy, loss, **MAE**, MSE, MAPE, and confusion matrix of DenseNet201.

DenseNet201 is a convolutional neural network consisting of 201 deep layers. The model is developed with the input, functional, and output layers as shown in Figure 9. The pooling layer is a fixed operation with no weighting factor [42]. Figure 10 shows the experimental results obtained using the DenseNet201 transfer model. The number of epochs is set to 50. The model shows that training accuracy is gradually increased from 30% to 90%. The increasing and decreasing validation accuracy attains a maximum 97% on the 13th epoch. The validation loss is around 1.2 in the 1st epoch and varies between 0.1 and 1.2. The validation loss attains a minimum value of 0.1 at epoch 50. The model shows a better fit where the training loss and validation loss both decrease and stabilize at a specific point [43]. The capability of the DenseNet201 **MAE** value is 0.02, the MSE value is 0.01, and the MAPE value is 0.01 for the test data. The confusion matrix shows that 37 samples are correctly predicted out of a total 40. Thus, overall accuracy is 93%.

The macro average precision of all the multiple classes and the weighted average precision attain 0.92 and 0.93 as shown in Table 4. The higher F1 scores among the multiple classes improve the model performance and the FL class has a maximum of 0.93. The F1 scores of all classes are balanced between precision and recall [44].

### 8.4. Performance Evaluation of Inceptionv3 Model

**Figure 11:**
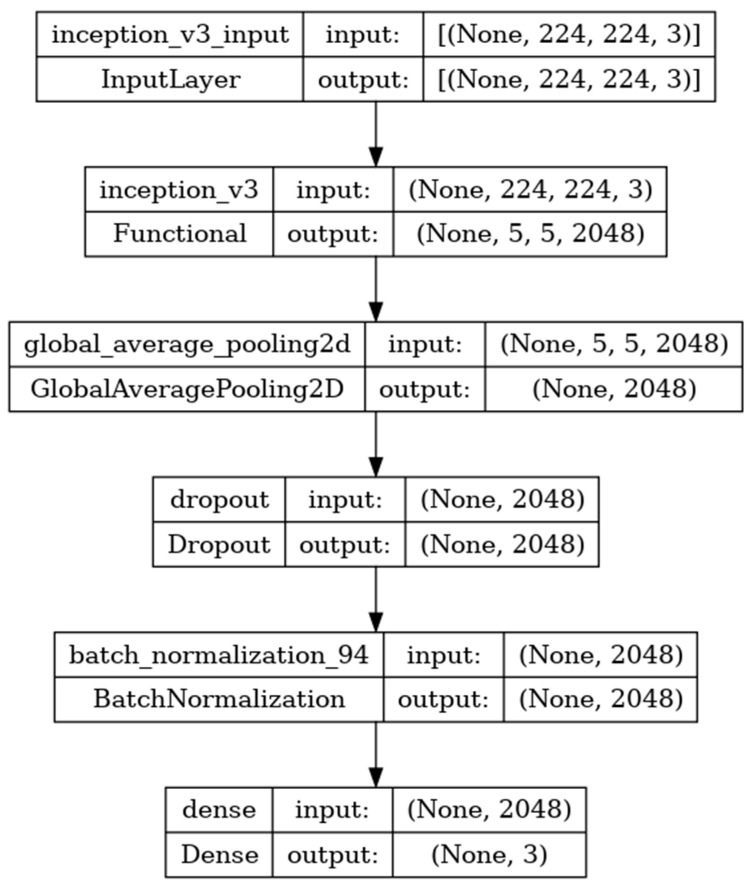
Inceptionv3 architecture summary.

**Figure 12:**
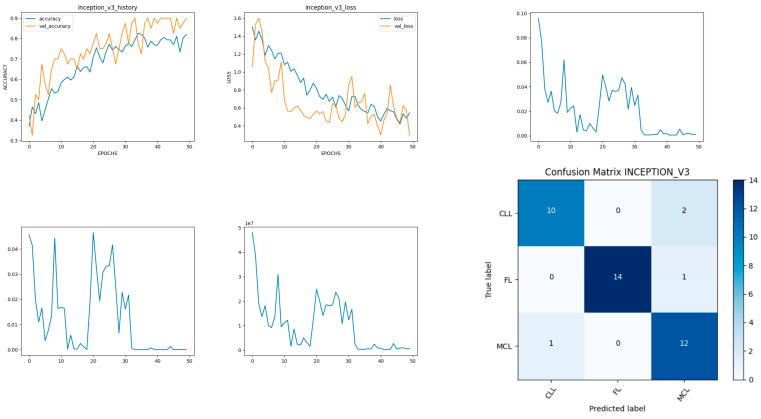
Accuracy, loss, **MAE**, MSE, MAPE, and confusion matrix of Inceptionv3.

Inceptionv3 is a deep-learning convolutional neural network image classification model [35]. The model is developed with the input, functional, and output layers as shown in Figure 11. The pooling layer is a fixed operation with no weighting factor [45]. Figure 12 shows the experimental results obtained using the Inceptionv3 transfer model. The number of epochs is set to 50. The model shows that training accuracy is gradually increased from 35% to 80%. The validation accuracy is slightly higher than the training accuracy for all epochs and attains a maximum 90% at the 50th epoch.

The validation loss is around 1.1 in the 1st epoch and decreases to 0.1 at the 50th epoch. The model shows good fit where the training loss and validation loss both decrease and stabilize at a specific point. The capability of the Inceptionv3 **MAE** value is 0.01, the MSE value is 0.01, and the MAPE value is 0.01 for the test data [46].

The confusion matrix shows that 36 samples are correctly predicted out of a total 40. Thus, overall accuracy is 90%. The macro average precision of all the multiple classes and the weighted average precision attain 0.90 and 0.91 as shown in Table 5. The higher F1 scores among the multiple classes improve the model performance and the FL class has a maximum of 0.97. The F1 scores of all classes are balanced between precision and recall [47].

### 8.5. Performance Evaluation of Xception Model

**Figure 13:**
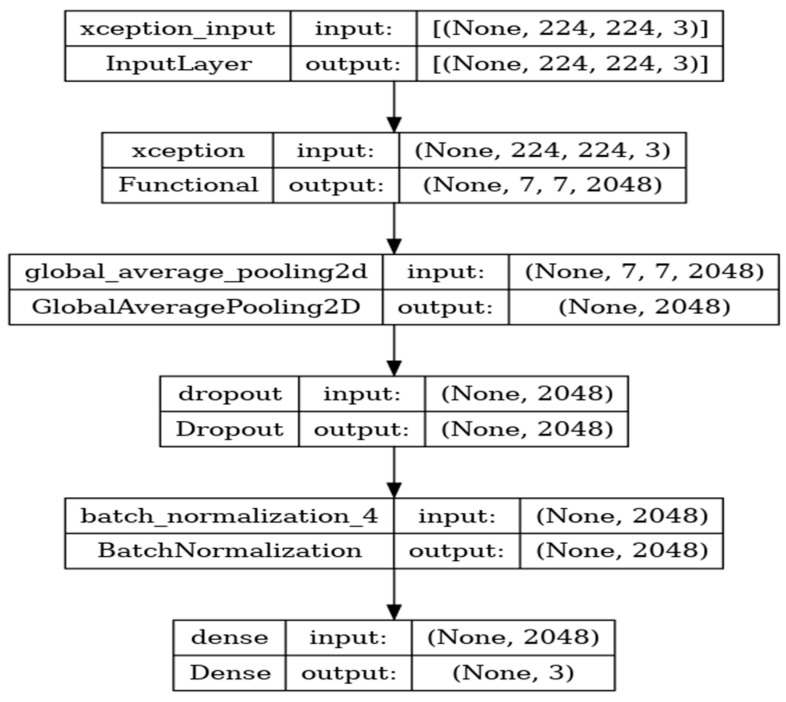
Xception architecture summary.

Xception is a deep-learning convolutional neural network of 71 layers. The model is developed with the input, functional, and output layers as shown in Figure 13.

**Figure 14:**
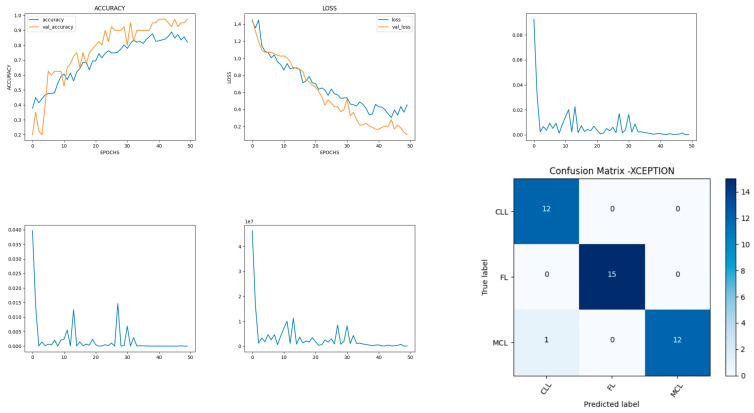
Accuracy, loss, **MAE**, MSE, MAPE, and confusion matrix of Xception.

The pooling layer is a fixed operation with no weighting factor. Figure 14 shows the experimental results obtained using the Xception transfer model. The number of epochs is set to 50. The model shows that training accuracy is gradually increased from 40% to 80%. The validation accuracy is slightly higher than the training accuracy for all epochs and attains a maximum 97% at the 50th epoch. The validation loss is around 1.4 in the 1st epoch and decreases to 0.1 at the 50th epoch. The model shows good fit where the training loss and validation loss both decrease and stabilize at a specific point [48]. The model performance is improved where training loss is slightly higher than the validation loss. The capability of the Xception **MAE** value is 0.01, the MSE value is 0.01, and the MAPE value is 0.01 for the test data.

The confusion matrix shows that 39 samples are correctly predicted out of a total 40. Thus, overall accuracy is 97%. The macro average precision of all the multiple classes and the weighted average precision values are 0.97 and 0.98 as shown in Table 6. The higher F1 scores among the multiple classes improve the model performance and the FL class has a maximum of 1.0. The F1 scores of all classes are balanced between precision and recall.

### 8.6. Performance Evaluation of Proposed Ensemble of Inceptionv3 and Xception Models

The ensemble architecture of InceptionV3 and Xception is developed to diagnose lymphoma cells using a new multi-cancer kaggle dataset which consists of 15,000 multi-class images. There are three types of ensemble techniques, namely bagging, boosting, and stacking. Each ensemble technique has its own merits and demerits; the stacking approach is a two–level classification technique and improves accuracy. Thus, the proposed system adopts a stacking-based ensemble approach to classifying lymphoma cancer cells.

**Figure 15:**
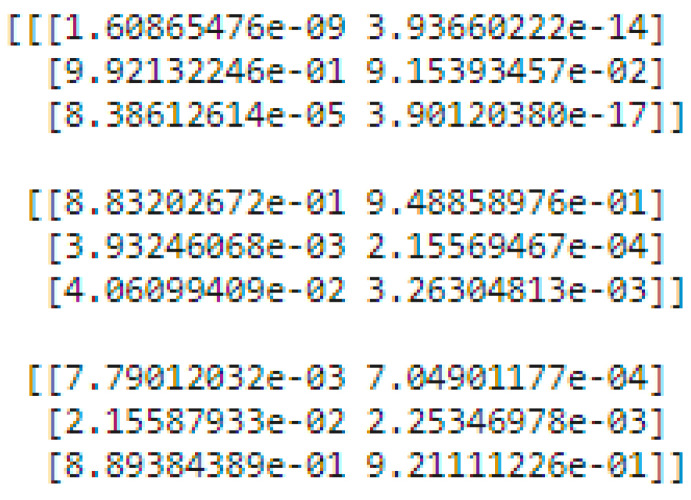
The proposed method level-0 classifier (Inception and Xception) output.

There are two levels of classification approach followed in the stacking method. On the first level, base or weak learners are used to predict the probabilities of each class. The predicted probabilities are then fed into the second-level classifier or Meta classifier to predict the final results (target value such as CLL, FL, and MCL). The proposed approach is adopted advanced deep network architectures as the base level classifiers such as Inception v3 and Xception. The base level classifiers then generate a matrix which contains the predicted probabilities of CLL, FL, and MCL images. The proposed approach used a CNN model for the Meta classifier. The generated matrix is then inputted to the Meta classifier or CNN model to be trained. Finally, the CNN can predict the lymphoma cancer categories such as CLL, FL, and MCL of the test set. The dataset contains three equal parts of 5000 for CLL, 5000 for FL, and 5000 for MCL. The 20 percent of 3000 test samples is split into 1029 samples of CLL, 962 samples of MCL, and 1009 samples of FL. All dataset images were in the JPEG file format with a size of 512 × 512 pixels. These two advanced deep-learning models produce the predicted probabilities of each class as the output. For each image, there are three probability values outputted by each level-0 classifier. The proposed method uses two deep-learning models such as Xception and Inceptionv3 in the level-0. So, for each image, six predicted probabilities are generated. In this way, for all the training images, predicted probabilities are generated that create a new dataset with six columns of predicted probabilities. A sample of the generated new dataset is shown in Figure 15. The output of the level-0 classifiers is given as the input for the level-1 classifier. For the Meta classifier, the CNN is used and the input for the CNN is the new dataset, which is generated by level-0 classifiers.

**Figure 16:**
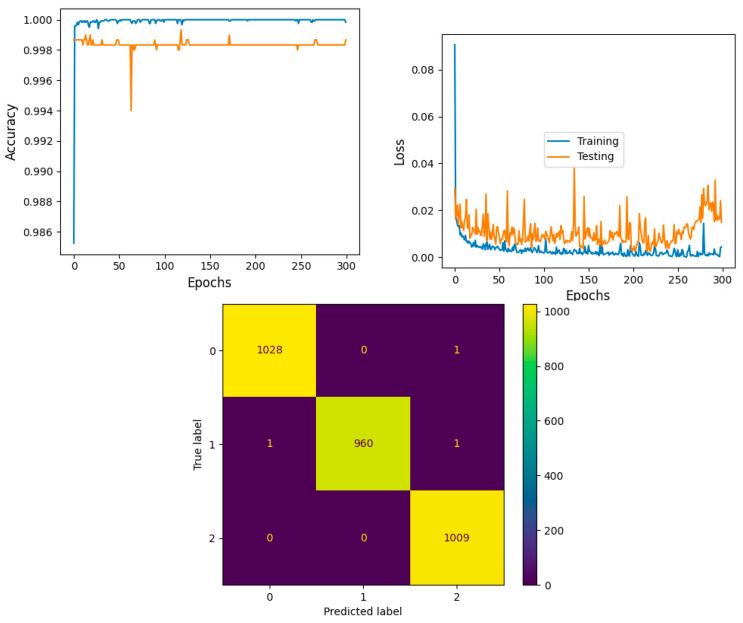
Accuracy, loss, and confusion matrix of ensemble model Inceptionv3 and Xception.

The model shows that training accuracy is increased to 95%. The validation accuracy is in line with the training accuracy for all epochs and attains a maximum 99% at the 300th epoch. The validation loss is around 0.08 in the 1st epoch and decreases to 0.1 at the 300th epoch. The model shows good fit where the training loss and validation loss both decrease and stabilize at a specific point. Thus, the ensemble model performance is improved and shown in Figure 16.

The confusion matrix shows that 2997 samples are correctly predicted out of a total 3000. The proposed stacked ensemble method prediction of CLL, FL, and MCL classes is explained in this section. For the CLL category, the correctly predicted test samples are 1028 out of 1029. For the MCL category, 960 test samples are correctly classified out of 962. For FL, all the test samples are correctly predicted and there are no incorrect samples. Thus, overall accuracy is 99%, which is shown in Table 7a. The higher F1 scores among the multiple classes improve the model performance. The F1 scores of all classes are balanced between precision and recall. A high sensitivity shows that the model is correctly identifying most of the positive results and the high value of specificity shows a higher value of true negatives. In Table 7b, the obtained results of sensitivity and specificity are shown.

## 9. Comparative Analysis of Non-ensemble and Proposed Ensemble Models

This section presents an approach for classifying the diagnosis of malignant lymphoma by applying non-ensemble models such as VGG16, VGG19, DenseNet201, Inceptionv3, and Xception. A stacked ensemble model (Inceptionv3, Xception, and CNN) is developed to improve the accuracy rate. The accuracy rate, precision, sensitivity, and F1 score are the indexes used to test and evaluate the performance of these models. The comparative experimental results are summarized in Table 8.

The average accuracy rate of this multi-classification lymphoma is the final performance evaluation index [49]. The accuracy of the Xception pre-trained network is higher than that of the other pre-trained networks, showing that Xception is a better classification non-ensemble model in the lymphoma multi-class images dataset. The proposed ensemble model had a higher testing accuracy of 99% greater than the efficient non-ensemble Xception model. It also attained the highest score for precision, recall, F1 score, and sensitivity metrics of 99% consistently compared with non-ensemble methods.

### Comparative Analysis of Proposed Ensemble Model with Prior Models

By comparing the performance of the proposed system to that of the previous systems, it is made evident that the proposed stacked ensemble model proposed in this study exhibits superior performance across all metrics. Hamdi et al. [21] developed a model for identifying the critical features for diagnosing WSI images of malignant lymphomas. The composite model combining the features of MobileNet-VGG16, VGG16-AlexNet, and MobileNet-AlexNet was developed using XGBoost and decision tree networks. The average accuracy of the proposed model was 96.2%. The performance measures of sensitivity, specificity, and precision are 96.5%, 97.8%, and 96.77%, respectively. Al-Mekhlafi et al. [50] proposed a two-hybrid systems model that utilized the FFNN classifier to classify images of malignant lymphomas from two datasets. In both malignant lymphoma datasets, the ResNet-50 + SVM network exhibits superior performance compared to the DenseNet-121 + SVM network. The overall accuracy rate of the model was 98.4 and the other performance measures such as sensitivity, specificity, and precision were 98.2, 98.4, and 98.5, respectively.

## 10. Conclusions and Scope for Future Research

The diagnosis of malignant lymphoma cells faces numerous challenges in distinguishing different classes, particularly during the early stages. Artificial intelligence supports physicians in distinguishing the classes of malignant lymphoma. In our work, the malignant lymphoma multi-class image datasets from various sources are trained using five pre-trained methodologies for diagnosing malignant lymphoma. The non-ensemble Convolutional Neural Network model is used to train the learning model with pre-trained weights VGG16, VGG19, DenseNet 201, InveptionV3, and Xception. The trained model is tested with the sample dataset and the predicted results match the trained data. DenseNet201, Inceptionv3, and Xception attain greater than 90% accuracy. These models are a good fit with minimum **MAE**, MSE, MAPE, and validation loss. The accuracies of VGG16 and VGG19 are very low and these are found to be overfitted models. Among the non-ensemble models, the Xception network outperformed all other models with 97% validation accuracy and minimum validation loss. Further, to improve the accuracy of the model, an ensemble architecture is proposed by using two efficient architectures, the Inceptionv3 and Xception models. The stacked ensemble model is trained and tested using the multi-cancer kaggle WSI image dataset. The stacked ensemble model exhibits higher testing accuracy of 99% greater than the Xception model. The proposed model has demonstrated that, it has achieved greater performance measures for precision, recall, F1 score, and sensitivity of 99%. There are still some misclassifications in our models. Further research is needed to improve the model’s performance using difficult cases and training using a multi-center database. The limitation of the proposed stacked ensemble technique is that no image feature extraction techniques are used to extract significant features that help the stacking model to make the classification output faster. The second limitation of the proposed model is that, the proposed method considered CLL, MCL, and FL categories of lymphoma for diagnosis. The sub categories of CLL, MCL, and FL is not considered in the presented work. The third limitation is that memory insufficiency was raised due to 15,000 Numpy array generation. The images are converted into Numpy array for the classification models for training and testing purposes. To overcome this problem, an image resizing approach is applied in the preprocessing step. Without this image resizing process, the proposed model and non-ensemble model outputs could be poor. In future work, a federated learning approach will be deployed to make the prediction from different computer systems. This feature will allow the system to take more input samples for the training and testing phases.

## Figures and Tables

**Table 1:** Transfer Learning Models for Malignant Lymphoma.

Model and Algorithm	Dataset	Performance	Literature
Combining MobileNet VGG16, VGG16-AlexNet, and MobileNet-AlexNet models. XGBoost and Decision Tree algorithm classifies using ant colony optimization (ACO).	Kaggle dataset of 15,000 WSI images with FL, CLL, and MCL of malignant lymphomas.	Accuracy: 95.3%, Precision: 95.77%, Sensitivity: 95.7%, Specificity: 96.8%	Hamdi et al., 2023 [21]
Faster R-CNN with pre-trained network.	A total of 1326 image samples are collected and trained from Ruijing Hospital, Shanghai Jiaotong University	Detection rate of lymphoma is higher than 96%	Sheng et al., 2020 [14]
Deep-learning framework, for follicular lymphoma (FL) diagnosis. Testing and validation performed using Bayesian neural networks (BNNs).	FL cases were extracted from the lymphopath database in two pathology departments (Toulouse University Cancer Institute and Dijon University Hospital, France).	The trained models generate accurate diagnosis with an Area Under the Curve reaching 0.99.	Syrykh et al., 2020 [22]
Diagnosed primary central nervous system lymphoma using CNN model via logistic regression integrated with multi-parametric MR images of PCNSL and GBM without tumor delineation.	Images extracted from Huashan Hospital, Fudan University, China, among 289 patients with PCNSL 139 and GBM 153.	The accuracy of DF-CNN model reaches 0.899 and greater than the other model IF-CNN value of 0.830	Xia et al., 2021 [23]
CNN used to differentiate Burkitt lymphoma (BL) and large B-cell lymphoma (DLBCL).	A total of 10,818 images of BL (*n* = 34) and DLBCL (*n* = 36) samples were used for training the CNN.	Samples are classified correctly to 94%. Receiver operating characteristic curve analysis area is 0.92 for both DLBCL and BL.	Mohlman et al., 2020 [24]
Deep residual neural network model (ResNet-50) with 374 lymphoma pathology images and classified by the softmax layer	Kaggle’s dataset of 374 pathology images are TIF format and includes CLL, FL, and MCL lymphoma.	The training result classification accuracy was 98.63% of GA-BP and BP neural network.	Zhang et al., 2021 [25]
U-Net Model. FOXP3+ Image segmentation framework for Biomarkers in Follicular Lymphomas. Biomarker segmentation is obtained using U-Net model.	BC Cancer Research Institute with high resolution images (2886 × 2886) without annotations.	The model was able to predict Positivity of FOXP3+ given a TMA core	Francisco et al. [26]
The ensembled classifier based on deep neural networks with eleven layers, four convolutional layers, and two fully connected layers.	Hematoxylin and eosin (H&E), 388 image samples extracted at Kurume University, Japan, from 2010 to 2017.	The classifier reaches the levels of accuracy of 94.0%, 93.0%, and 92.0% for multi-class image patches.	Miyoshi et al. 2020 [9]
ResNet-101 pre-trained network is used to extract image features of lymphoma cells.	The microscopic blood image dataset with 1673 image samples of leukocytes is modified by their types of lymphoma, blasts, lymphocytes.	The proposed system reaches 98.74% precision in lymphoma classification and 99.22% precision for lymphoma image cell extraction.	Reena et al., 2022 [27]
Predictive model of Classification trees using Python script and Pandas for data pre-processing and SciKit learn for training and validation	The data collected from A.O. Ordine Mauriziano, Turin (Italy).	Overall accuracy of 92.68%, sensitivity of 88.54%, specificity of 98.77%.	Gaidano et al., 2020 [11]
Automatic detection of the MYC translocations in DLBCL using deep learning model	HE-stained glass slides from 157 patients with DLBCL that were analyzed using FISH	Classification accuracy reaches 0.67. Under the ROC curve, the accuracy was 0.77 with sensitivity 0.88 and specificity 0.66.	Swiderska et al., 2020 [28]

**Table 2:** Performance Results of VGG16.

Classification Report (VGG16)	Precision	Recall	F1 Score
CLL (Chronic Lymphocytic Leukemia)	0.00	0.00	0.00
FL (Follicular Lymphoma)	0.38	1.00	0.55
MCL (Mantle Cell Lymphoma)	0.00	0.00	0.00
Accuracy	-	-	0.38
Macro Average	0.12	0.33	0.18
Weighted Average	0.14	0.38	0.20

**Table 3:** Performance Results of VGG19.

Classification Report (VGG19)	Precision	Recall	F1 Score
CLL (Chronic Lymphocytic Leukemia)	0.50	0.42	0.45
FL (Follicular Lymphoma)	0.50	1.00	0.67
MCL (Mantle Cell Lymphoma)	0.00	0.00	0.00
Accuracy			0.50
Macro Average	0.33	0.47	0.37
Weighted Average	0.34	0.50	0.39

**Table 4:** Performance Results of DenseNet201.

Classification Report (DenseNet201)	Precision	Recall	F1 Score
CLL (Chronic Lymphocytic Leukemia)	0.92	0.92	0.92
FL (Follicular Lymphoma)	0.93	0.93	0.93
MCL (Mantle Cell Lymphoma)	0.92	0.92	0.92
Accuracy			0.93
Macro Average	0.92	0.92	0.92
Weighted Average	0.93	0.93	0.93

**Table 5:** Performance Results of Inceptionv3.

Classification Report (Inceptionv3)	Precision	Recall	F1 Score
CLL (Chronic Lymphocytic Leukemia)	0.91	0.83	0.87
FL (Follicular Lymphoma)	1.00	0.93	0.97
MCL (Mantle Cell Lymphoma)	0.80	0.92	0.86
Accuracy			0.90
Macro Average	0.90	0.90	0.90
Weighted Average	0.91	0.90	0.90

**Table 6:** Performance Results of Xception.

Classification Report (Xception)	Precision	Recall	F1 Score
CLL (Chronic Lymphocytic Leukemia)	0.92	1.0	0.96
FL (Follicular Lymphoma)	1.00	1.0	1.0
MCL (Mantle Cell Lymphoma)	1.00	0.92	0.96
Accuracy			0.97
Macro Average	0.97	0.97	0.97
Weighted Average	0.98	0.97	0.97

**Table 7:** Performance results of stacked ensemble model.

(**a**)				
**Classification Report** **(Stacked Ensemble Technique)**	**Precision**	**Recall**	**F1 Score**	**Specificity**
CLL (Chronic Lymphocytic Leukemia)	0.99	0.99	0.99	0.99
FL (Follicular Lymphoma)	1.0	1.0	0.99	0.99
MCL (Mantle Cell Lymphoma)	0.99	0.99	0.99	1
Accuracy			0.99	
(**b**)				
**Class**	**Sensitivity**		**Specificity**	
CLL (Chronic Lymphocytic Leukemia)	0.999494		0.997073	
FL (Follicular Lymphoma)	1.000000		1.000000	
MCL (Mantle Cell Lymphoma)	0.998504		0.998995	

**Table 8:** Performance evaluation of non-ensemble and stacked ensemble models.

Pre-Trained Models	Average Accuracy %	Average Precision %	Average Recall %(Sensitivity)	Average F1 Score
VGG16	38	40	38	20
VGG19	50	34	50	40
DenseNet201	93	93	93	93
Inceptionv3	90	91	90	90
Xception	97	97	97	97
Proposed Stacked Ensemble Model (Inceptionv3 + Xception + CNN)	99	99	99	99

## Data Availability

The multi cancer kaggle dataset of malignant Lymphoma images used in this study donloaded from the https://www.kaggle.com/datasets/andrewmvd/malignant-lymphoma-classification & https://www.kaggle.com/datasets/obulisainaren/multi-cancer (accessed on 12 December 2023).
